# Incidence of accidents and injuries in children under 6 years old in southern Iran: a population-based study

**DOI:** 10.5249/jivr.vo112i2.1280

**Published:** 2020-07

**Authors:** Alireza Mirahmadizadeh, Abdolrasool Hemmati, Soraya Zahmatkesh, Masoomeh Saffari, Pezhman Bagheri

**Affiliations:** ^*a*^ Non-Communicable Diseases Research Center, Shiraz University of Medical Sciences, Shiraz, Iran.; ^*b*^ Vice-Chancellor of Health Affairs, Shiraz University of Medical Sciences, Shiraz, Iran.; ^*c*^ Department of Community Medicine, Shiraz University of Medical Sciences, Shiraz, Iran.; ^*d*^ Student Research Committee, Shiraz University of Medical Sciences, Shiraz, Iran.

**Keywords:** Incidence, Injuries, Accidents, Child

## Abstract

**Background:**

Accidents and injuries are the leading cause of childhood morbidity and mortality. This study aimed to investigate the incidences of different causes of accidents in children under 6 years old.

**Methods:**

This population-based cross-sectional study was carried out in one year (2016-2017) on a sample of 6000 children in Southern Iran with a multistage sampling method using a standard checklist for comprehensive child health monitoring.

**Results:**

The annual incidence rate of accidents was 16% and the mean age of accident victims was 2.5±1.5 years. Of these, 17.3% and 14.8% were male and female, respectively. 25% of the children suffered from more than one accident. The most common causes of accidents were burns (16%), falls (14%), and accidents involving objects (10%). In multivariate analysis, a higher number of male children in the family and lower child age significantly increased the likelihood of accidents (p less than 0.05).

**Conclusions:**

This study was a direct survey of the population, and showed that the incidence rate of accidents in children in southern Iran was in line with that of other regions of Iran, but less than the world average. There was no significant difference in accident etiology and only trends in etiology were found compared to studies using existing data.

## Introduction

As defined by the World Health Organization (WHO), injury, normally resulting from an accident, is a random, preventable and unavoidable event, and is a product of acute exposure to environmental or physical forces.^1^ Today, more than ever, accidents and injuries resulting from technological advancements and changes in modern-day lifestyles^2^ threaten the lives of people in different parts of the world.^3^ Accidents and injuries are among the most significant causes of mortality in children, as the most vulnerable group worldwide.^4^ At the moment, accidents involving children have become a public health problem and with a direct and indirect economic burden on societies.^5^

Studies in the United States indicate an annual incidence of 2,800 deaths due to domestic injuries in children, resulting in 13 million children having at least one outpatient treatment and 74,000 children being admitted to hospital.^6,7^ A 2008 WHO report entitled "child injury prevention" shows that mortality rates from unintentional injuries in children in low-income countries are 3.4 times than those in higher-income and more-developed countries (41.7 million compared to 12.2 million).^8^

A comprehensive study covering six European countries in 2009 reports an annual incidence rate of accidents occurring in 23% of children.^9^ A regional study in India in 2018 estimated the annual incidence of unintentional events in children to be 16.6%.^10^ A 2011 study in Saudi Arabia reported the incidence in children to be 22.2%.^11^ In other studies in 2018^12^ and 2019,^13^ the incidence rates of unintentional injury were reported to be 16.2 and 62.7 per 100000 respectively. Also, a new study published in 2019^14^ reported that over a 10-year period, the annual incidence rates of unintentional injury among children under the age of 1 and between 1-5 years old increased by 1.7% and 1.5%, respectively. In the search for a pattern, a global-scale study in low- and middle-income countries found falls and traffic accidents to be the most commonly-reported incidents in children.^15^ Previous studies in Iran have shown falls and traffic accidents,^16^ swallowing foreign objects, burns, and poisoning^17,18^ as well as electrical injury, falling over, drowning and blunt trauma^19^ to be some of the most significant events in children. In Iranian children aged 1-14, injuries and accidents accounted for 16.6% of deaths, with traffic accidents (37.5%), drowning (17.9%) and burns (12.1%) considered the most prevalent accidents in this age group.^20^

Injuries and accidents in children have high economic and social consequences. Further, reducing the mortality rate in this vulnerable group is one of the major goals of the Millennium Development.^20,21^ Important factors such as physiological constraints, the process of growth and development, sensory and motor development, behavioral characteristics, and reactive capacity (experience, the need for education, surveillance, adventure, and risky behaviors) make this group vulnerable to accidents.^4,22^

Considering the importance and consequences of accidents in children as a productive group in society, together with there being a higher proportion of injuries compared with other diseases in this group, planning is necessary to prioritize the prevention of various types of incidents in children. Conversely, major studies carried out in this area in Iran and other countries have been based on the statistics of the relevant organizations or on existing data. To date, almost no population-based statistics or surveys have investigated the incidence of accidents either in children or in adults. This study aimed to investigate the incidence of accidents in children under 6 years old in the form of a provincial survey in Fars Province of Iran.

## Methods 

**Study method and sampling **

This study was a descriptive-analytic study conducted in Fars province, South Iran, during a one-year period in 2016-2017. In the current study, the target population included all children under 6 years of age (n= 377,202), covered by health centers of Shiraz University of Medical Sciences. A random sample was selected as the study population as following. Initially, all the counties of the province and all health centers (urban bases) in towns, and health houses in villages were randomly considered as classes and clusters, respectively. From this, households were selected according to the inclusion criteria of having a child under the age of 6 and a willingness to respond. According to the literature review, the estimated effect size (incidence of accidents) was estimated to be 5.5%.^9,23-25^ Using a 95% confidence interval and an estimated error of 1%, a sample size of 1997 people was calculated. According to the cluster sampling method, the design effect was considered to be 1.5. Thus, with a coefficient of 3, the final sample size was calculated to be 6,000 people.

In each household, the mother was interviewed. For sampling, based on the urban and rural population, the ratio of urban and rural areas was 60% and 40%, respectively. As such, a cluster was defined as 30 children under the age of 6 years. Therefore, a total of 200 clusters were needed, divided according to the ratio of the cities to villages, indicated 83 and 117 clusters for rural and urban areas, respectively. Primary schools in cities and health houses in the villages were considered as cluster centers, and sampling was started from the right side of each center and continued until the sample size had been achieved.

To collect data, a researcher-made checklist was used to examine the health of any children in the household under the age of 6. This comprised of the child’s demographic data, including age, gender, place of residence, parental age, parental education and occupation, and ethnicity. Questions about accidents were designed according to the Iran’s Ministry of Health's standard checklist, and covered whether or not the child had been the victim of an accident in the year running up to the interview, as well as the type of accident, the location of the accident and the actions taken in the last year. This study was based on a project that was carried out as part of the routine periodic evaluation by the Deputy of Health Affairs to assess health in children living in Fars province. Therefore, the project was approved by the Health System Research (HSR) Committee and the Deputy Of Health Affairs of Shiraz University of Medical Sciences. All mothers were asked if they were willing to participate. All data was confidential and the data were analyzed for governmental purposes as well as for research. 

**Statistical methods **

The data were used to calculate the incidence of accidents in children in the year running up to the study. All data were analyzed using SPSS version 22 by conducting two-sample t-student tests, a one-way ANOVA and chi-square tests to analyze inter-group comparisons. A logistic regression was performed for modeling the causes of accidents. All results are reported at a significant level of p≤0. 05. 

## Results

**Descriptive results**

Of the 6,000 children visited, the information of 270 children could not be collected, and as such, those children were removed from the sample. Data from the remaining 5,730 children were evaluated. The mean age of children was 1.98 ± 1.5 years, 51.9% of the children were male and 48.1% female. 57.4% of children lived in the city and 42.6% in the rural areas. In total, 16% of children incurred an accident in the year leading up to the study. 

The mean age of the accident group was 2.5 ± 1.5 years. The largest number of accidents occurred in children under 1-year-old, and the lowest incidence at 6 years old. Descriptive characteristics of the sample are given inTable 1 and 2.

**Table 1:** descriptive characteristics of the continuous variables.

Variable	Accident	Frequency	Means ± SD	Overall mean	p-value
**Mother's age**	Yes	915	30.2 ± 5.6	30.6 ± 7.6	0.04
	No	4777	30.7 ± 7.9		
**Father’s age**	Yes	912	35.1 ± 6.6	35.4 ± 10	0.27
	No	4768	35.4 ± 10.6		
**Number of family male**	Yes	758	1.23 ± .7	1.28 ± .7	0.01
	No	3750	1.30 ± .7		
**Number of family female**	Yes	683	1.23 ± .8	1.30 ± .8	0.001
	No	3636	1.31 ± .8		
**Birth weight (g)**	Yes	206	3156 ± 512.5	3166.1 ± 502.5	0.59
	No	1001	3168.2 ± 500.6		
**Weight during questioning (g)**	Yes	776	13694.6 ± 5224.2	12644.3 ± 5646.7	<0.001
	No	4284	12454 ± 5699.8		
**Child's age (month)**	Yes	169	30.4 ± 18.1	23.7 ± 18.4	<0.001
	No	930	22.5 ± 18.2		

**Table 2:** Distribution of descriptive variables and incident rate by descriptive variables.

Variable	Levels	Frequency (%)	Incidence rate (%) of accident	p-value
**Gender**	Male	51.9	17.3	0.009
	Female	48.1	14.8	
**Mother's literacy**	Illiterate	3.5	13.5	0.08
	Elementary	16.3	13.6	
	Middle	17.6	18	
	Diploma	41.1	16.5	
	College Education	21.5	15.9	
**Father’s literacy**	Illiterate	2.6	17.8	0.94
	Elementary	12.8	16.3	
	Middle	27.2	16	
	Diploma	37.3	15.6	
	College Education	20.2	16.4	
**Mother's job**	No	92.3	15.9	0.26
	Yes	7.6	17.2	
**Father’s job**	Unemployed	4	20.8	0.07
	Employed in public sector	15.1	14.7	
	Employed in private sector	6.7	19	
	Free	73.8	15.5	
**Mother's ethnicity**	Fars	72	16.7	0.38
	Lor	12.5	13.8	
	Turk	10.9	14	
	Kurd	0.2	20	
	Baloch	0.03	-	
	Arab	1.7	15.3	
	Non Iranian	2.6	17.3	
**Father’s ethnicity**	Fars	72.7	16.5	0.24
	Lor	12.3	13.3	
	Turk	10.3	15.4	
	Kurd	0.3	30	
	Baloch	0.03	-	
	Arab	1.6	15.1	
	Non Iranian	2.7	17	
**Being alive parents**	Yes	99.4	16	0.75
	Mother's death	0.1	28.6	
	Father’s death	0.4	20	
	Both died	0.2	20	
**Parents being together**	Yes	98.7	16.1	0.41
	No	1.3	12.5	
**Buy toys in recent 1 year**	Yes	89.7	16	0.6
	No	10.3	16.8	
**Type of delivery**	Natural	43.8	15.9	0.8
	Section	56.2	16.1	
**Place of delivery**	Public hospital	83.1	15.7	0.11
	Private hospital	15.2	16.6	
	Maternity facility center	0.6	15.6	
	Home	0.4	17.4	
	Others	0.7	31	
**Incident due to neglect of parents**	Yes	9.8	62.2	<0.001
	No	90.2	12.5	
**Household monthly income (Million Tomans)**	<1.5	75.3	16	0.88
	1.5-3	22.7	15.5	
	3-5	1.9	17.9	
	5-10	0.1	25	
	>10	0.041	-	
**Birth rank**	1	42.7	17	0.02
	2	36.4	16.2	
	3	15.1	14	
	4	4	11.7	
	5	1.2	21.5	
	6	0.4	36.4	
	7	0.1	12.5	
	8	0.03	-	

**Figure 1:**
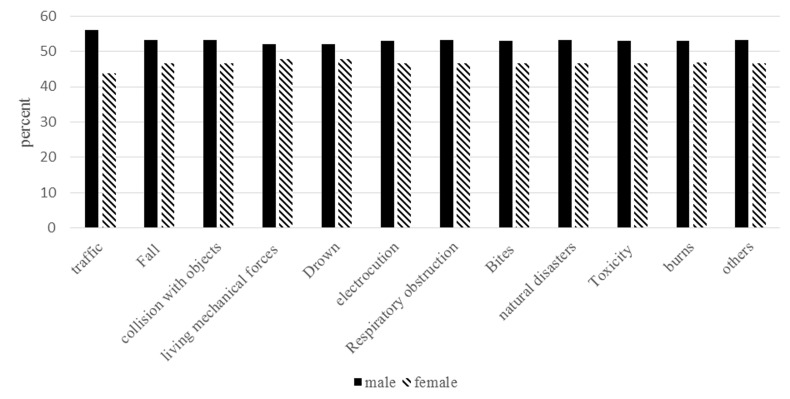
Distribution of accidents by gender.

In terms of the location of the accident, the incidence was higher in rural areas (16.2%) compared to cities (15.9%). There was a higher incidence of accidents reported in males (17.3% compared to 14.8% in females; p=0.56). The most common accident among males was traffic accidents and among females was mechanical forces and drowning (Fig.1).

**Figure 2:**
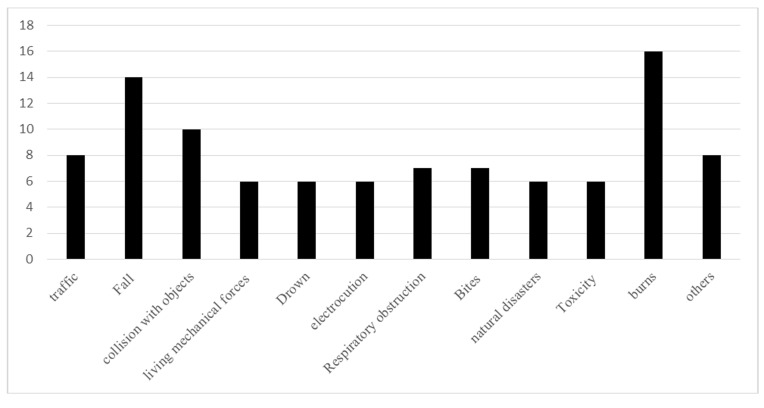
Total frequency of accidents.

In total, burns (16%), falls (14%), and collision with objects (10%) were the most numerous causes of accidents (Fig 2). Most accidents took place inside the house (77.15%; Table 3). The most common action to take following the accident was referring to physicians and health centers (34%).

**Table 3:** Distribution of accidents by Place of accident.

Accident type	Inside the house	Outdoor	No injury
**Transportation and traffic**	3.3	21.1	75.6
**Fall**	59.1	11.1	29.8
**Collision with objects**	43.5	9.6	46.9
**Collision with the living mechanical forces**	1.6	1.6	96.7
**Drown**	0.8	1.2	98
**Electrocution**	2.1	-	97.9
**Respiratory obstruction**	15.2	0.8	84.1
**Burns**	24.7	3.5	71.7
**Bites of animals and poisonous plants**	7.1	2.4	90.6
**Natural disasters**	1.3	-	98.7
**Toxicity**	4.2	1.3	94.6
**Others**	19.1	5	75.8

**Analytical Results**

In the univariate model, the binary logistic regression revealed that the number of male children in the family and the child's age were the main predictors of accidents (Table 4), such that for each male child in the household, the likelihood of an accident occurring in that year increased by 52.5%. Also, for each year of the child’s age, the likelihood reduced by 2%. The place of residence, parental age, educational level and occupation, ethnicity, cause and place of accident did not show significant relationships with the incidence of accidents. 

**Table 4:** logistic regression model results for predictor variables.

Variable	B	SE	WALD	DF	SIG.	EXP(B)	CI for (OR)	
							lower	upper
**Intercept **	1.663	0.430	14.964	1	0.000	5.277	-	-
**Number of male**	0.422	0.208	4.131	1	0.042	1.525	1.015	2.291
**Number of female**	0.197	0.169	1.364	1	0.243	1.218	.875	1.697
**Gender**	-0.057	0.279	0.042	1	0.838	0.944	.546	1.633
**Age**	-0.017	0.007	5.843	1	0.016	0.983	.969	.997
**Weight **	0.000	0.000	0.693	1	0.405	1	-	-

## Discussion

These results showed that the annual incidence of accidents in children was 16%. In order of frequency, burns, falls and collisions with objects were the most common causes. 25% of the children with accident history, experienced more than once in the past year. The most common location of the accident was inside the house, with the majority of accidents taking place in villages and in male children. The number of male children in the household and the child's age were the main predictors of the likelihood of an accident occurring.

Child health is a major indicator of societal health. Conversely, to improve child safety, it is necessary to study the patterns and causes of accidents in children. The rapid advancement of a variety of technologies, and children's ease of access to them have caused death and disability from accidents.^26^ Children also cannot understand the dangers of their surroundings and are more vulnerable to many risks.^27^

Accident statistics in Iran are mostly reported at the provincial level, and there are no accurate documented statistics of the national prevalence of accidents and injuries in children. Conversely, the present study uses a population-based approach and as such is more accurate and reliable compared to existing studies, which have been conducted based on organizational records and show relatively different trends.^28^

In Iran, several studies have been conducted on the incidence rate of accidents in children, and various statistics have been reported, as follows: 8% in Qazvin,^29^ 40% in Ahwaz,^17^ 25% and 1.2% in Fars,^30,31^ 23.3% in Kermanshah^32^ and 37% in Hamadan.^33^ The age range of the samples was different in these studies. which may account for the difference in incidence rate of the samples reported. The same variation can be seen in other countries: 19.4% in Ireland,^34^ 16.16% in India^10^ and 22.2% in Saudi Arabia.^11^ These statistics may vary due to cultural and socioeconomic differences, such as environmental health conditions, environmental safety standards, access to play equipment and learning, parental awareness, household income level, parental education and environmental physical structures. As such these do not provide a direct comparison with Iranian statistics. On the other hand, in all these studies, registered data were used no population-based data.

In the present study, as mentioned, 25% of the children with accident history, experienced more than once in the past year. It follows that there was a number of children who had been affected by more than one cause of an accident in one year. This has not previously been reported in the literature.

Burns, falling, and collisions with objects, were the most frequently reported causes of accidents in the present study, in line with previously published Iranian studies in this area.^4,16,17,26,29,35,36^ The overarching trends confirm current understanding, whereas more subtle differences may be associated with differences in the location, time and demographic characteristics of previous study samples. As such, judgments and comparisons must be made with caution. In this regard, the incidence of accidents should be considered highly dependent on the demographic characteristics of the children sampled. For example, it seems that children under five years are at a higher risk of burns, poisoning, and falling, while children older than 5 years are at higher risk for accidents in the street. In the majority of international studies, similar patterns are seen.^8,11,15,37-39^

In terms of age, there is a downward trend in incidence from children aged less than a year up to 6 years. This trend has been seen in almost all similar studies. Moreover, the occurrence of accidents in younger children has often been higher and with greater severity. Age, like gender and social class is closely associated with the prevalence of accidents in children, not only among Iranian studies but also in international studies.^34^ According to the available evidence, the severity of the negative consequences of accidents in children under 1 year old is quite different from children with 10 years of age. Male children compared to female children are more likely to incur all causes of accidents and their outcomes, and there is a large discrepancy of mortality rate in children by economic and social classes. Even in terms of burden of disease, studies have claimed that 13% of accidents are responsible for the burden of childhood disease, especially in young male children.^8,11,29,40-44^ Further, there are studies reporting that the risk of accidents in children increases with increasing age, such as Dal Santo et al.^39^ But most evidence is in line with higher incidence and severity in young children.

In our study, the most common causes of accidents in male children were traffic accidents and in female children were collisions with living mechanical forces as well as drowning. Most available evidence, including the studies conducted by Balan et al.^8^ , Bartelett^37^ and Fatmi,^45^ report high incidence of burns and heat-related accidents in females in terms of gender segregation of accidents; which differs from the pattern reported in our study in female children. In existing studies, the collaboration and participation of girls from a young age in activities such as making food and working in the kitchen, due to various cultural and gender reasons, are among the reasons for the high prevalence of this type of accident in female children. Also, female children’s clothing that is more susceptible to burning than male’s.^8^ Conversely, a higher incidence of accidents such as traffic accidents and falling in males can also be due to their higher freedom and free movement in the outside environment and riskier behavior compared to female children. In our study, one of the reasons for the occurrence of accidents such as drowning or dealing with the living mechanical forces has been the dominant pattern of agriculture and traditional life and the higher extent of rural areas among other cultural factors. Theoretically, as noted in Tabibi's study,^26^ the incidence of accidents in children belonging to lower social and economic classes is expected to be higher.^8^ The difference in children from middle-income families is likely due to changes in income levels that can be attributed to different socioeconomic classes over a 10-year period in Iran. 

In our study, the most common accident location was the home and the highest number of accidents occurred in the rural areas. In terms of higher incidence of accidents in rural areas, some studies agree with our results, whereas others claim that the prevalence of accidents in urban areas is higher.^18,20,22,26,46^ The lack of recreational infrastructure for children in rural areas, along with other economic, cultural and geographical factors, makes the rural environment more susceptible to accidents compared to the urban environment. Therefore, rural and urban segregation of accidents does not necessarily implicate a greater or smaller number of accidents in these areas; rather, different patterns of events in these areas. Concerning the occurrence of accidents inside the house, there are contradictory findings depending on the age, gender, and setting of the study; the majority of studies identified the site of the accident through a descriptive approach, similar to that used in the present study, whereas other studies^21,22,47^ reported the place of most accidents within the home or outside^10,44,48^ the home. 

The number of male children in the household and the child's age were identified as significant predictors for accidents. It seems that determining the causes of accidents in children is largely dependent on the study setting, when the study was conducted, and the objectives of the research; as different causes of accidents have been identified depending on the research objectives of different studies. In Hosseinzadeh,^29^ Tabibi,^26^ Siran He,^15^ Mathur ^10^ et.al and a study in Riyadh,^11^ respectively, age and seasonal trends, parental education, age and sex of a child, age and rural location and proximity to public spaces and playing in the street have been identified as factors contributing to the incidence rate of accidents in children.

The most common action following the occurrence of an accident was found to be referral to physicians and health centers. It seems that the most important and most common action following an injury is referral to a health center, which, in turn depends on the severity of the consequences and the availability of health services; a finding that is independent whether or not a population-based study was conducted.

Finally, the most important strength of this study is the adequate sample size determined through sample-size calculation. The use of large-scale (provincial) demographic information and the population-base methodology allows for the study of accidents that that will provide reliable outcomes for use by policymakers. There are some weaknesses of the study, which make the generalization of the study slightly more difficult. The lack of specific statistics on intentional and unintentional accidents, the lack of insight into mortality rates, social class and time of accident as well as domestic violence, alcohol consumption or the use of illicit drugs by the mother or father, child abuse, household overcrowding and substandard living arrangements, prospectively collected data, ethical or religious differences, history of violence in family, or mortality resulting from injuries are some of the more important limitations of this study. As such, including these factors is recommended in future studies.

## Conclusion

In this study, the incidence of accidents in this region (Southern Iran) is lower than the global average. In contrast to other regions of Iran, the area had comparable incidence rate. Fully characterizing the incidence of various causes of accidents in this vulnerable and significant group at a demographic level requires numerous population-based studies to be conducted in different populations of Iranian children and in different locations. It is suggested that designing and conducting population-based studies in children and adults on accident rates will help to create a realistic picture of the accidents in different parts of Iran.

**Acknowledgement**

We have to express our sincere thanks to all the mothers who, in the province, have been caring and sincere with the researchers of the project to provide accurate information about their children, as well as respectable Rebecca S. Dewey, University of Nottingham, UK for her kind help in language edition and interviewers who tirelessly and steadfastly lead to the outcome of this project. 
